# *Staphylococcaceae* isolates from the reef macroalga *Sargassum ilicifolium*, found in the coastal waters of Singapore

**DOI:** 10.1128/mra.00335-25

**Published:** 2025-06-18

**Authors:** Prasha Maithani, Jessica Alison Taylor, Su Xuan Gan, Dalong Hu, Joao Paulo Andre Pereyra, Rebecca Josephine Case

**Affiliations:** 1 Singapore Center for Environmental Life Sciences Engineering (SCELSE), Nanyang Technological University (NTU) 54761, Singapore, Singapore; 2 School of Biological Sciences, Nanyang Technological University (NTU) 219572, Singapore, Singapore; 3 Singapore Center for Environmental Life Sciences Engineering (SCELSE), National University of Singapore (NUS) 37580, Singapore, Singapore; 4 Saw Swee Hock School of Public Health, National University of Singapore (NUS) 37580, Singapore, Singapore; University of Southern California, Los Angeles, California, USA

**Keywords:** macroalga, *Sargassum ilicifolium*, *Staphylococcaceae*, antimicrobial resistance, Singapore

## Abstract

Two strains belonging to the family *Staphylococcaceae* (SSi009 and SSi170) were isolated from *Sargassum ilicifolium*. Genome sequencing identified several antimicrobial mechanisms within both organisms’ genomes, revealing their potential to act as gene reservoirs of antimicrobial resistance in the environment.

## ANNOUNCEMENT

*Mammaliicoccus* (previously *Staphylococcus*) and *Staphylococcus* are genera within the family *Staphylococcaceae* (1, 2). These taxa are found in domestic animals and waterways such as beaches and estuaries (2–4). Their genomes commonly harbor several antimicrobial-resistance (AMR) and virulence genes (5, 6), making them critical in the monitoring of AMR worldwide.

Thalli of the brown macroalga *Sargassum ilicifolium* were collected from St. John’s Island, Singapore (1.222353 N 103.848702 E), pressed onto enrichment agar (2.5% [wt/vol] BBL brain heart infusion broth, 1.5% [wt/vol] Instant Ocean sea salt, 0.03% [wt/vol] K_2_TeO_3_, and 1% [vol/vol] Tween 80) (7), and incubated at 30°C for 24 h. Plates were subcultured from individual colonies to ensure purity. Single colony isolates were inoculated for 24 h in liquid and solid enrichment media, biomass combined, washed in phosphate-buffered saline solution (PBS), and concentrated.

Genomic DNA was extracted from 35 mg biomass using the DNeasy Blood and Tissue Kit (Qiagen, Hilden, Germany), following the manufacturer’s protocol for gram-positive bacteria, with the addition of RNase A (Qiagen) (8). Library preparation was done using the TruSeq Nano DNA Library Prep Kit (Illumina, San Diego, USA), and sequencing was performed using the Illumina HiSeq X Ten platform for 150 bp paired-end reads at the SCELSE sequencing facility, NTU.

All reads were trimmed and quality-filtered using Trimmomatic version 0.39 (9). Default parameters were used for all software unless specified. Genomes were assembled using Shovill version 1.1.0 and SPAdes version 3.15.5 (10), and then annotated using the Prokaryotic Genome Annotation Pipeline version 6.7 (11). Assembled genomes (Table 1) were checked for plasmids using PlasmidFinder version 2.0.1 (12) and Staramr version 0.9.1 (13). Plasmids with a rep13 and rep19c origin of replication were found in SSi009 and SSi170 (Singaporean *S. ilicifolium* isolate), respectively. The rep19c-containing plasmid in SSi170 encoded erythromycin and azithromycin resistance genes.

**TABLE 1:** Genome features and GenBank accession numbers of the two *Staphylococcaceae* isolates

Isolate	Genome size (bp)	No. of CDS^*a*^	No. of rRNAs	No. of tRNAs	G+C content (%)	No. of contigs	*N*_50_ (bp)	*L*_50_	Assembly accession no.	SRA accession no.	No. of reads	Average read length (bp)	Mean coverage
*Mammaliicoccus sciuri* SSi009	2,847,455	2,775	7	59	32.6	44	302,036	3	JBMIHW000000000	SRX28012226	9,461,482	151	2,706.47
*Staphylococcus arlettae* SSi170	2,510,479	2,391	11	61	33.3	59	217,966	4	JBMIHV000000000	SRX28012227	6,628,937	151	961.955

^*a*^ CDS, coding DNA sequences.

Isolates were identified using the Genome Taxonomy Database Toolkit (GTDB-Tk) version 1.7.0 (14), characterized by topology and average nucleotide identity (ANI). The highest similarity for SSi009 and SSi170 was to *Mammaliicoccus sciuri* (GCF002901825) and *Staphylococcus arlettae* (GCF002902345). Digital DNA-DNA hybridization (dDDH) values were also determined between isolates and reference genomes using the Genome-to-Genome Distance Calculator, version 3.0 (15). The SSi009 isolate had ANI and dDDH scores of >95% and <70% to its GTDB-Tk identified reference genome, while the SSi170 isolate had ANI and dDDH scores of >95% and >70% to its reference genome.

Further analysis using BlastKOALA (16) for KEGG Orthology assignments revealed that both genomes contained the complete gene set for antimicrobial resistance mechanisms: (i) cationic antimicrobial peptide (CAMP) resistance (*graS*, *graR*, and *mprF*) (17) and (ii) efflux pumps (*abcA*, *mgrA*, and *norB*) (18, 19). Two additional mechanisms were identified in *S. arlettae* SSi170: (i) beta-lactam resistance (*bla*) system (*blaR1*, *blaI*, and *blaZ*) (20) and (ii) the CAMP resistance dltABCD operon (*graS*, *graR*, *dltA*, *dltB*, *dltC*, and *dltD*) (21). The presence of several AMR mechanisms in these genomes indicates their role as AMR gene reservoirs (5, 6).

## Data Availability

The SRA data were deposited to NCBI, and the complete genome sequences were deposited to GenBank under accession numbers listed in Table 1. BlastKOALA assignments were uploaded to FigShare and are accessible under the DOI 10.6084/m9.figshare.29069714.
